# Mechanisms of Antibiotic Failure During *Staphylococcus aureus* Osteomyelitis

**DOI:** 10.3389/fimmu.2021.638085

**Published:** 2021-02-12

**Authors:** Brittney D. Gimza, James E. Cassat

**Affiliations:** ^1^Division of Pediatric Infectious Diseases, Department of Pediatrics, Vanderbilt University Medical Center, Nashville, TN, United States; ^2^Department of Pathology, Microbiology, and Immunology, Vanderbilt University Medical Center, Nashville, TN, United States; ^3^Vanderbilt Center for Bone Biology, Vanderbilt University Medical Center, Nashville, TN, United States; ^4^Department of Biomedical Engineering, Vanderbilt University, Nashville, TN, United States; ^5^Vanderbilt Institute for Infection, Immunology, and Inflammation (VI4), Vanderbilt University Medical Center, Nashville, TN, United States

**Keywords:** *Staphylococcus aureus*, osteomyelitis, antibiotic failure, biofilm, SCVs, persisters, intracellular survival, antibiotic tolerance

## Abstract

*Staphylococcus aureus* is a highly successful Gram-positive pathogen capable of causing both superficial and invasive, life-threatening diseases. Of the invasive disease manifestations, osteomyelitis or infection of bone, is one of the most prevalent, with *S. aureus* serving as the most common etiologic agent. Treatment of osteomyelitis is arduous, and is made more difficult by the widespread emergence of antimicrobial resistant strains, the capacity of staphylococci to exhibit tolerance to antibiotics despite originating from a genetically susceptible background, and the significant bone remodeling and destruction that accompanies infection. As a result, there is a need for a better understanding of the factors that lead to antibiotic failure in invasive staphylococcal infections such as osteomyelitis. In this review article, we discuss the different non-resistance mechanisms of antibiotic failure in *S. aureus*. We focus on how bacterial niche and destructive tissue remodeling impact antibiotic efficacy, the significance of biofilm formation in promoting antibiotic tolerance and persister cell formation, metabolically quiescent small colony variants (SCVs), and potential antibiotic-protected reservoirs within the substructure of bone.

## Introduction

*Staphylococcus aureus* is the leading cause of osteomyelitis, which is defined as inflammation of bone but is most commonly encountered in the setting of bacterial infection. Osteomyelitis can result in significant morbidity such as progressive bone damage, pathologic fractures, and septicemia (1, 2). Bone infections typically develop via three clinical mechanisms, including hematogenous seeding of bone, invasion of bone from a contiguous source (e.g., following trauma or via spread from soft tissues), or infection occurring secondary to vascular insufficiency or neuropathy (e.g., diabetic foot infection) (1). Osteomyelitis can be isolated to a single part of the bone or it can impact multiple regions including the bone marrow, cortical and trabecular bone, the periosteum, and surrounding soft tissues (1, 2).

**Figure 1:**
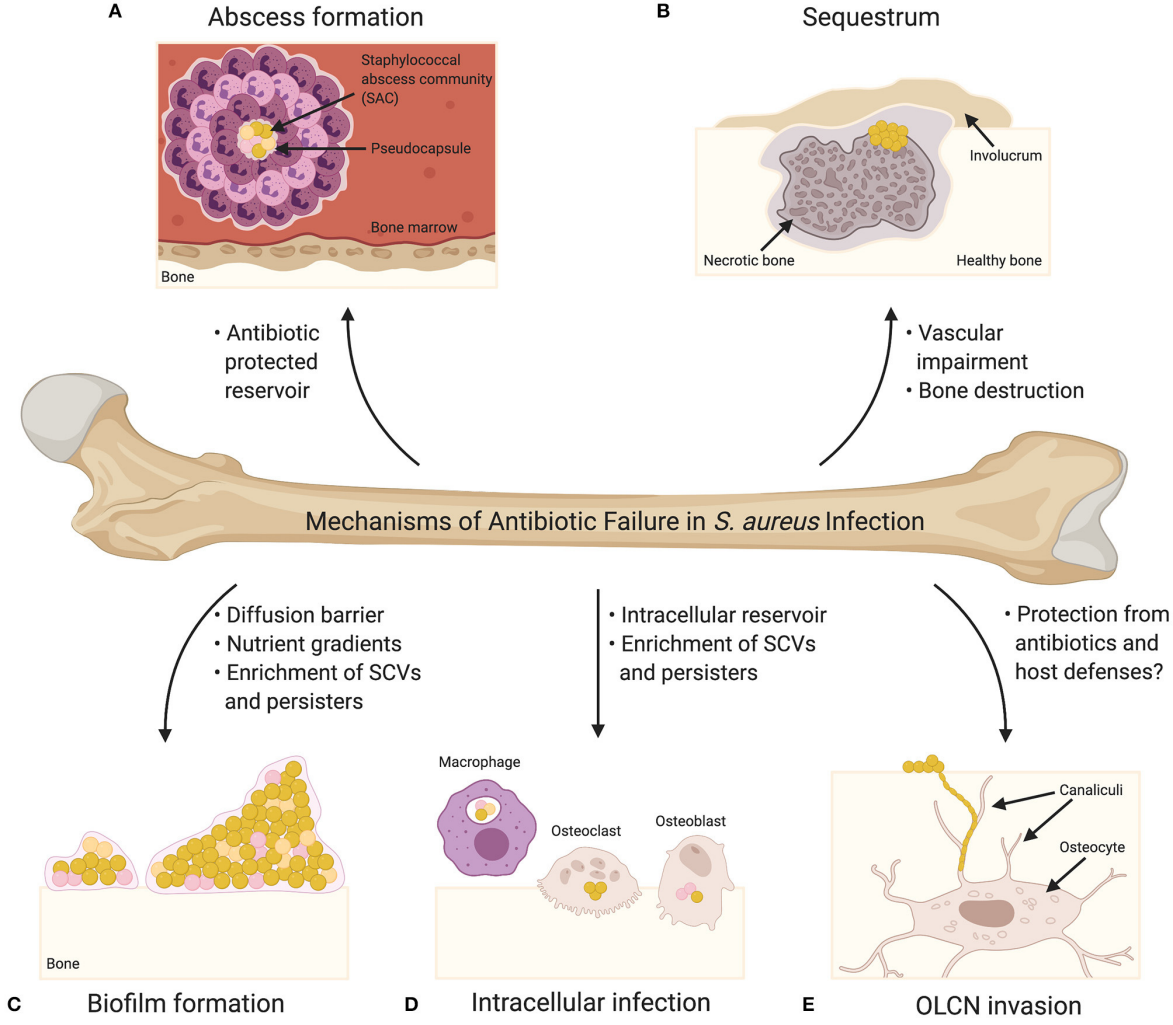
Mechanisms of Antibiotic Failure in *S. aureus* Infection. **(A)** Abscesses are the characteristic tissue lesions of invasive staphylococcal infection. The bacteria within the core of abscesses are referred to as staphylococcal abscess communities (SACs), which are surrounded by a pseudocapsule made of fibrin and other host extracellular matrix proteins. The SAC is surrounded by immune cells, including both viable and non-viable neutrophils. Bacteria within a SAC exhibit increased tolerance to antibiotic treatment. **(B)** Abscess formation and exuberant inflammation during osteomyelitis compromise the blood supply to the bone leading to bone necrosis. Necrotic bone fragments result in the formation of tissue lesions known as sequestra, which are characteristic of chronic osteomyelitis and serve as a nidus for persistent infection. In response to the sequestrum, new bone formation occurs resulting in the formation of a pathologic lesion known as an involucrum. Vascular impairment resulting from infection significantly diminishes the effectiveness of systemic antibiotics. **(C)** Biofilm formation on bone greatly contributes to bacterial persistence during bone infection, and biofilm-associated bacteria exhibit increased tolerance to antibiotics. Biofilms may act as diffusion barriers for antibiotics, thereby reducing the penetrance of antibiotics toward the deeper layers of the biofilm. The biofilm environment, which is characterized by significant nutrient and oxygen gradients, is thought to promote the production of antibiotic tolerant bacterial cells (e.g., small colony variants [SCVs] and persisters) (SCVs are illustrated as pink cocci; persisters are illustrated as orange cocci). **(D)** *S. aureus* has been shown to invade and survive within professional phagocytes (e.g., macrophages) and resident bone cells (e.g., osteoclasts and osteoblasts). Intracellular survival contributes to antibiotic tolerance given that most antibiotics act extracellularly, and the intracellular host environment is thought to enrich the formation of SCVs and persisters. **(E)** Osteocytes, the major cell type embedded within the bone matrix, reside in structures known as lacunae, and connect to one another via a three-dimensional network of channels known as canaliculi. Colonization of the osteocyte lacuno-canalicular network (OLCN) is believed to promote chronicity of *S. aureus* osteomyelitis as the antibiotic concentrations needed for bacterial eradication may not be possible to achieve within the infected OLCN. Bacteria within the OLCN might also be protected from the host response.

The treatment of acute osteomyelitis using antibiotic therapy is associated with a high success rate (3); however, many cases require surgical debridement in addition to antibiotic therapy and despite these measures, treatments fail in ~20% of cases (4). Osteomyelitis treatment is complicated by a number of factors, including: (1) widespread antimicrobial resistance, (2) antibiotic tolerance as a result of metabolic changes and/or biofilm formation, (3) the inability of antibiotics to penetrate infected and damaged bone, and (4) the colonization of potentially antibiotic-protected reservoirs within the substructure of bone. Accordingly, *S. aureus* has multiple mechanisms outside of traditionally defined antibiotic resistance that can contribute to treatment failure of osteomyelitis infections, and as such, these mechanisms (Figure 1) will be the focus of this review.

## The Development of Chronic Osteomyelitis

Key characteristics of osteomyelitis are severe inflammation, vascular impairment, and localized bone loss and destruction (2). The host responds to the presence of bacteria such as *S. aureus* by releasing inflammatory factors and degradative enzymes from immune cells, which contribute to the destruction of bone matrix and bone trabeculae (1, 5–10). Many of the innate immune responses involved in antibacterial host defense also have significant impacts on bone homeostasis, and the release of inflammatory mediators at the infection site can result in decreased osteoblast-mediated bone formation and increased osteoclast activation and bone resorption, thereby promoting bone loss. To counteract the host immune response, *S. aureus* releases specific immunoevasive virulence factors, including those that have been linked to osteomyelitis pathogenesis such as protein A (Spa) and the major histocompatibility complex (MHC) class II analog protein (Map) (10). In addition to the primary role of protein A in immune evasion, Spa has also been documented to contribute to staphylococcal osteomyelitis by altering bone homeostasis via direct interactions with osteoclasts and osteoblasts, resulting in bone loss (11, 12). Map contributes to osteomyelitis by altering T-cell function (13).

*S. aureus* immunoevasive factors also contribute to the formation of abscesses, which are the characteristic tissue lesions of invasive staphylococcal infection and consist of a three-dimensional community of bacteria surrounded by immune cells. This physical segregation of bacterial cells from the surrounding host tissue is predicted to protect pathogens from both the host response and antibiotic treatment (14–16). The bacteria within the core of abscesses are referred to as staphylococcal abscess communities (SACs), which are surrounded by a pseudocapsule made of fibrin and other host extracellular matrix proteins (14, 16). In addition, this dense community of bacteria is surrounded by immune cells, including both viable and necrotic neutrophils. For a more detailed description of the mechanisms underlying staphylococcal abscess formation, readers are directed to the outstanding review by Cheng et al. (14). During osteomyelitis, abscesses commonly form within the bone marrow space as well as in the surrounding soft tissues (15, 17, 18). Abscess formation and exuberant inflammation during osteomyelitis also compromise the blood supply to the bone leading to further bone necrosis. Necrotic bone fragments result in the formation of lesions known as sequestra, which are characteristic of chronic osteomyelitis and serve as a nidus for persistent infection (1). In response to the sequestrum, new bone formation occurs resulting in the formation of a pathologic lesion known as an involucrum (10). With regards to treatment failure, it is hypothesized that the SACs play an important role given that the bacteria have an increased tolerance to antibiotic treatment (16). Further, Hofstee et al. revealed that the upon mechanically dispersing SACs, bacteria were efficiently killed, suggesting the pseudocapsule provides protection from antibiotic treatment. Two staphylococcal coagulases, staphylocoagulase (Coa) and von Willebrand factor-binding protein (vWbp), are important for the formation of the pseudocapsule (14) and therefore could play an important role in the treatment failure of *S. aureus*. Additionally, as a result of the vascular impairment in infected bone, systemic antibiotics are thought to be significantly less effective (1).

## The Role of Biofilm Formation

*S. aureus* biofilm formation on necrotic bone and implanted material greatly contributes to bacterial persistence during bone infection, and is presumed to be a leading cause of treatment recalcitrance during chronic osteomyelitis (19, 20). Biofilms are multicellular microbial communities encased within a self-produced matrix that are formed on either organic or inorganic surfaces and exhibit increased tolerance to antibiotics (21–25). Vascular impairment and decreased oxygen tension within sequestra provide ideal conditions which promote the attachment of planktonic bacteria and ultimately biofilm formation (2). Regardless of how the bacteria reach bone or implant surfaces, the bacteria attach to the surfaces using microbial surface components recognizing adhesive matrix molecules (MSCRAMMs). Specifically, the colonization of bone occurs through the attachment of planktonic bacteria to extracellular matrix proteins, bone cells, or plasma proteins (26–28). For example, two staphylococcal adhesins that play an important role in bone adhesion during osteomyelitis are collagen adhesion protein (Cna) and bone sialoprotein (Bbp) (29–31). Following attachment, bacteria produce an extracellular matrix (ECM) composed of proteins, polysaccharides, and/or extracellular DNA (eDNA), leading to the formation of a mature biofilm (32). The extracellular matrix is important for binding bacterial cells to each other and to the substrate, as well as for maintaining the biofilms structural integrity. In addition, protection is provided to bacteria within the biofilm given the decreased susceptibility of the biofilm to the host immune response, environmental stresses, and antibiotics (33).

Bacteria within biofilms have been found to be 10 to 1,000 times more tolerant to antibiotic treatment in comparison to the genetically identical planktonic bacteria (34). Biofilms may act as diffusion barriers for antibiotics thereby reducing the penetrance of antibiotics toward the deeper layers of the biofilm (34). However, the biofilm diffusion barrier function cannot solely account for the dramatic reduction in antibiotic susceptibility observed, as antimicrobials that do not interact with components of the ECM are able to diffuse at a rate comparable to diffusion through water (35). As such, the contribution of the diffusion barrier to the overall increased tolerance of biofilms is likely less important for some antibiotics, and in these cases altered metabolic activity of biofilm bacteria is hypothesized to be a major driver of antibiotic tolerance.

### Small Colony Variants and Persisters

Small colony variants (SCVs) and persisters are two phenomena reflective of the altered metabolic activity of bacteria within biofilms (36). Persisters are dormant phenotypic variants with increased antibiotic tolerance, found within a susceptible bacterial population (37). These antibiotic-tolerant cells are transient variants which revert to a drug susceptible state upon subculturing in fresh growth media (38). The biofilm environment, which is characterized by a paucity of nutrients and oxygen, is thought to promote the production of persisters given that these conditions support a reduction in metabolic activity and a low energy state—traits of antibiotic tolerant persisters (39). SCVs are characterized by their pinpoint colony size, altered pigmentation, slow growth, activation of the stringent response, downregulation of virulence genes via a reduction of the *agr* quorum sensing system, and upregulation of genes associated with adhesion and biofilm formation through the activation of the alternative sigma factor B (*sigB*) (40–42). A variety of different stressors have been shown to trigger SCVs, including antibiotic pressure, low pH, limited nutrients, cationic peptides, reactive oxygen species, and intracellular localization (43, 44). Specific environmental stressors can result in the production of phenotypically distinct SCVs which can be transient variants that will revert back to wild-type under favorable conditions, or irreversible SCVs that result from permanent genetic changes (45–50). In the context of osteomyelitis, SCVs have been isolated from chronic bone infections and are believed to support persistent and relapsing infections (51, 52).

The increased antibiotic tolerance observed with persisters and SCVs is suggested to be a result of their altered metabolic activity (15, 47, 53, 54). With regard to SCVs, mutations associated with the production of these variants most commonly occur in menadione and hemin biosynthesis genes (55). Importantly, menadione and hemin are essential in the biosynthesis of menaquinone and cytochromes which are components of the electron transport chain. Consequently, ATP production decreases as a result of a reduction in membrane potential, resulting in the slowing of bacterial growth. Given that bactericidal antibiotics target active cellular processes, a decrease in growth rate can result in increased tolerance to these antibiotics (41, 47). In addition, the decreased membrane potential can reduce the influx of aminoglycoside antibiotics, resulting in decreased susceptibility to these antibiotics (55).

While persisters are similar to SCVs in that they tolerate antibiotic treatment by entering into a more metabolically quiescent state, the specific mechanism of persister formation in *S. aureus* remained relatively unclear until recently. In *Escherichia coli*, persister formation is linked to toxin-antitoxin modules; however, when this was investigated in *S. aureus* it was found that the deletion of these modules did not influence the levels of persisters (56). However, the same study revealed that the formation of persisters is associated with a stochastic entrance into stationary phase and the depletion of intracellular ATP. As such, the decrease in ATP results in a reduction in growth rate, and therefore a reduction in the targets of many antibiotics, resulting in an increase in antibiotic tolerance. Most recently, in an effort to identify specific metabolic pathways resulting in persister formation, Zalis et al. found that, within a growing population, there are cells which stochastically express enzymes of the tricarboxylic acid (TCA) cycle at low levels, resulting in decreased ATP production and ultimately an increase in antibiotic tolerance (57).

## Intracellular Survival of *S. aureus*

An additional mechanism potentially contributing to antibiotic tolerance in the setting of invasive infection is the intracellular survival of *S. aureus*. Previous studies have demonstrated the capacity of *S. aureus* to invade and survive within professional phagocytes including macrophages and neutrophils, as well as non-phagocytic cells such as epithelial cells, keratinocytes, endothelial cells, fibroblasts, and bone cells (58–62). Following internalization, bacteria are able to escape cell death by evading lysosomal compartments, preventing phagolysosomal fusion, or persisting within vacuoles (63, 64). It has been shown that *S. aureus* is able to not only survive within the phagolysosome but also initiate intracellular replication (65). *S. aureus* is also thought to persist intracellularly by adopting a metabolically inactive state similar to SCVs (48). Intracellular persisters in macrophages have also been identified following antibiotic exposure, suggesting that the intracellular environment could contribute to *S. aureus* persistence and relapsing infections (66). A more recent study found that macrophages are unable to efficiently kill *S. aureus* and that tolerance is induced to multiple antibiotics in response to exposure to reactive oxygen species, thus highlighting a more direct contribution of intracellular survival to antibiotic tolerance (67).

With regards to osteomyelitis, *S. aureus* has been shown *in vitro* to infect skeletal cells, including osteoblasts (68–70) and osteocytes (71). Additionally, *S. aureus* has been observed residing within osteoclasts *in vitro* and *in vivo* (72). Notably, using TRAP-tdTomato reporter mice with a green fluorescent protein (GFP)-expressing *S. aureus* strain, Krauss et al. were able to image calvarial histological sections using confocal microscopy, and GFP-expressing *S. aureus* were localized within TRAP-tdTomato osteoclasts (72). However, the contribution of intracellular survival in the context of human osteomyelitis remains unclear, as thus far it has been difficult to rigorously document intracellular communities of bacteria in histologic specimens.

Although the contribution of intracellular survival in osteomyelitis is not entirely understood, the effects of antibiotics on intracellular survival remain of significant interest to the research community. A study by Ellington et al. found that following long-term *S. aureus* survival within osteoblasts, bacterial sensitivity to antibiotic treatment decreases (73). *S. aureus* survival in osteoblasts is believed to occur partly as a result of SCV formation, and SCVs increase following the treatment with select antibiotics (74–76). In addition to osteoblasts, SCVs have also been shown to form upon internalization by terminally differentiated osteocytes (71). Given the increased antibiotic tolerance observed with SCVs, their intracellular presence would further complicate treatment. Furthermore, if the intracellular survival of *S. aureus* contributes to the establishment of chronic infections, antibiotic treatment could inadvertently promote infection persistence by promoting SCV formation.

## Colonization of the Osteocyte Lacuno-Canalicular Network (OLCN)

Colonization of the osteocyte lacuno-canalicular network is an additional mechanism considered to promote persistence during *S. aureus* osteomyelitis. Osteocytes, the major cell type embedded within the bone matrix (77), create a three-dimensional network in which osteocytes directly connect individual lacunae via canaliculi (78). Lacunae are the spaces containing the individual osteocytes, whereas the canaliculi are the channels containing the osteocyte cytoplasmic processes (79). Recently, *S. aureus* was found to invade the OLCN in a murine model of osteomyelitis (80). When imaging live cortical bone using transmission electron microscopy (TEM), chains of individual cocci were present within canaliculi. Given the non-motile nature of *S. aureus*, it is hypothesized that the bacteria are accessing the network and moving throughout via asymmetric binary fission. Colonization of the OLCN was further confirmed with a human *S. aureus* diabetic foot infection where the use of TEM identified cocci within the osteocyte lacunar and canalicular space (81). This discovery suggests a novel mechanism of persistence in chronic osteomyelitis as the bacteria within the OLCN might be protected from the host response and the bone matrix could serve as a nutrient source further supporting long-term survival. Importantly, the minimal inhibitory concentrations needed for antibiotic therapies may not be possible to achieve within an infected OLCN (81).

## Targeting Antibiotic Tolerance Mechanisms to Improve *S. aureus* Treatment

A greater understanding of the aforementioned tolerance mechanisms and their contribution to antibiotic failure is facilitating the development of more effective treatment strategies. In the context of osteomyelitis, one approach is the improved targeting of *S. aureus* within host cells given that most antibiotics do not freely diffuse across the cell membrane. To assist in improving osteomyelitis treatment, Valour et al. determined the effectiveness of frontline antimicrobials by assessing their impact on intraosteoblastic *S. aureus* and the emergence of SCVs (75). This group found that some antibiotics (i.e., vancomycin and daptomycin) have no significant impact on intracellular bacterial growth whilst only ofloxacin had both strong intracellular activity and a limiting effect on SCV emergence. This study emphasizes that in refining the antimicrobial therapy for osteomyelitis, the intraosteoblastic activity of antibiotics should be considered. A combinatorial treatment approach consisting of an anti-biofilm compound (i.e., rifampin) with an effective intracellular-acting compound may be more effective. Two additional studies working toward targeting intracellular bacteria both leveraged engineering approaches to enhance the effectiveness of peptidoglycan hydrolases, which are highly specific bactericidal enzymes, as a treatment for *S. aureus* infections (82, 83). These enzymes were modified to contain either protein transduction domains or cell-penetrating peptides, both of which facilitate entry into mammalian cells and ultimately resulted in the enhanced eradication of intracellular staphylococci in osteoblasts. Multiple studies have also focused on the use of nanoparticles to improve the treatment of intracellular bacteria in osteoblasts and osteoclasts (84–86). One study in particular investigated the use of hybrid nanoparticles to improve the delivery of rifampicin to the intracellular environment (86). Using rifampicin-loaded nanoparticles, Guo et al. increased the delivery of rifampicin within osteoblasts as well as decreased the number of surviving bacteria following treatment. Two additional studies have reported the use of silver nanoparticles to reduce bacterial survival within osteoclasts and osteoblasts (84, 85). Specifically, Aurore et al. determined that with the use of silver nanoparticles, the decrease in bacterial recovery from osteoclasts correlated with an increase in reactive oxygen responses (84).

Another approach to improve *S. aureus* treatment is the targeting of persisters and SCVs by restoring uptake of aminoglycosides, which is normally prevented by the reduced membrane potential of these cells. A study by Radlinski et al. found that rhamnolipids, a biosurfactant produced by *Pseudomonas aeruginosa*, were able to improve the effectiveness of the aminoglycoside tobramycin against *S. aureus* (87). Ultimately, it was shown that the increased uptake of tobramycin was PMF-independent, and this resulted in inhibition of otherwise tolerant bacterial populations such as persisters, SCVs, biofilm, and anaerobic populations of *S. aureus* (88).

Lastly, efforts are now being focused on identifying novel drug targets that are critical for *S. aureus* invasion into the OLCN network. Using a microfluidic silicon membrane canalicular array (μSiM-CA) developed to model *S. aureus* invasion of the OLCN, Masters et al. screened select transposon mutants and were able to identify penicillin binding protein 4 (PBP4) as critical to OLCN invasion (89). In a murine model of implant-associated osteomyelitis, a strain lacking PBP4 displayed a decreased tolerance to vancomycin treatment, a reduction in pathogenic bone-loss at the implant site, and an inability to invade and colonize OLCN. As such, given the significant contribution of PBP4 to deep bone invasion, the development of a PBP4-specific inhibitor could improve osteomyelitis antimicrobial therapies. Taken together, these studies highlight opportunities to increase the efficacy of traditional antibiotics by leveraging adjunctive treatments that target intracellular pathogens, persisters, and niche-protected bacteria.

## Conclusion

*Staphylococcus aureus* osteomyelitis remains a serious health threat given the significant morbidity and treatment recalcitrance of these infections. *S. aureus* not only is able to adapt to changing host environments and evade the host immune response, but it also has multiple mechanisms to promote tolerance to antibiotic treatment. As a result, treatment of osteomyelitis requires long term antibiotic therapy, often in combination with surgical debridement which can further increase osteomyelitis morbidity. In order to improve the outcome of osteomyelitis treatment and reduce the risk of relapse, a greater understanding of the tolerance mechanisms used by *S. aureus* to survive antibiotic treatment is essential. Furthermore, when developing novel treatment strategies, it should be considered that the effectiveness of treatments *in vitro* in a clinical microbiology setting may not be an appropriate representation of effectiveness *in vivo*.

## Author Contributions

JC: conceptualization, writing—reviewing and editing, supervision, and funding acquisition. BG: writing—original draft preparation and visualization. Both authors contributed to the article and approved the submitted version.

## Conflict of Interest

The authors declare that the research was conducted in the absence of any commercial or financial relationships that could be construed as a potential conflict of interest.
